# Assessing Plasma C-Peptide Levels and Their Relationship with Health-Related Quality of Life in Patients with Prediabetes and Type 1 and Type 2 Diabetes

**DOI:** 10.3390/biomedicines13102423

**Published:** 2025-10-03

**Authors:** Sajid Iqbal, Silvia Reverté-Villarroya, Nayab Batool Rizvi, Hira Butt, Josep Lluís Clúa-Espuny

**Affiliations:** 1 Nursing Department, Universitat Rovira i Virgili, Campus Terres de l’Ebre, 43500 Tortosa, Spain; iqbal.sajid@estudiants.urv.cat; 2 Research Group on Advanced Nursing (CARING)-161, Universitat Rovira i Virgili, 43002 Tarragona, Spain; 3 School of Chemistry, University of the Punjab (PU), Lahore 54590, Pakistan; 4 Primary Health-Care Center EAP Tortosa Est, Institut Català de la Salut, CAP El Temple Plaça Carrilet, 43500 Tortosa, Spain; jlclua@telefonica.net; 5 Ebrictus Research Group, Research Support Unit Terres de l’Ebre, Institut Universitari d’Investigació en Atenció Primària Jordi Gol (IDIAPJGol) (Barcelona), Terres de l’Ebre, 43500 Tortosa, Spain

**Keywords:** health-related quality of life, EQ-5D-5L, EQ-VAS scores, β-cell function, C-peptide, diabetes mellitus, prediabetes, type 1 diabetes, type 2 diabetes

## Abstract

**Background/Objectives:** Understanding the relationship between plasma connecting peptide (C-peptide) levels and health-related quality of life (HRQoL) can inform diabetes management strategies. This study aimed to assess plasma C-peptide levels, HRQoL, and their association in patients with prediabetes, type 1 diabetes (T1D), and type 2 diabetes (T2D) attending outpatient departments (OPDs) in tertiary care hospitals. **Methods**: A cross-sectional survey was conducted between 1 January and 30 June 2023, using the EuroQoL Five Dimensions (EQ-5D-5L) instrument. Participants with prediabetes, T1D, or T2D were recruited from OPDs in diabetology, endocrinology, general practice, and family medicine at Sheikh Zayed Hospital (SZH) and Mayo Hospital (MH) in Pakistan. Plasma C-peptide levels were measured and HRQoL was assessed using EQ-5D-5L and the EQ Visual Analog Scale (VAS). **Results**: A total of 301 patients were included: 42 with prediabetes (14%), 70 with T1D (23.2%), and 189 with T2D (62.8%). The median C-peptide level was 0.46 nmol/L (IQR 0.13–0.85), the HRQoL score was 78.5% (IQR 63.2–100%), and the EQ VAS score was 85% (IQR 70–90%). C-peptide levels were significantly correlated with HRQoL scores (r = 0.14, *p* < 0.02) and differed across mobility, daily activity, pain/discomfort, and anxiety/depression domains (all *p* < 0.02). HRQoL scores significantly varied among the three groups (*p* < 0.0001), particularly in the aforementioned domains. **Conclusions**: C-peptide levels and HRQoL differ significantly across diabetes types, with lower C-peptide associated with reduced mobility, increased pain, and mental health issues. These findings underscore the importance of targeting C-peptide regulation to enhance HRQOL in diabetic populations.

## 1. Introduction

The C-peptide, a byproduct of insulin production, is essential for understanding pancreatic function and managing diabetes [1,2,3]. Assessing C-peptide levels provides valuable insights into the residual beta-cell function in patients with diabetes, which is significantly linked with both the physiological and psychological aspects of the disease [4,5]. In T1D and T2D, C-peptide concentrations vary, reflecting distinct challenges and trajectories in disease progression and management. These variations in C-peptide levels can be profoundly linked to the HRQoL for patients, highlighting the importance of tailored approaches to treatment and support [1,6].

HRQoL is an important outcome measure in chronic disease management and is conventionally assessed using validated questionnaires such as the Short Form 36 Health Survey (SF-36) [7], World Health Organization Quality of Life-BREF (WHOQOL-BREF) [8], and EQ-5D-5L [9]. These instruments capture multiple dimensions of well-being, including physical function, mental health, social relationships, and environmental conditions. According to the World Health Organization’s (WHO’s) European Health Equity Status Report, achieving sustainable health outcomes requires more than just access to medical care, and it depends on income security, decent housing, social capital, and employment opportunities, all of which influence HRQoL. In individuals with diabetes, HRQoL is often compromised due to disease-related complications, glycemic fluctuations, and treatment burden, which contribute to physical discomfort and psychological distress [10].

Within this framework, C-peptide may serve as a critical biological mediator of HRQoL. In individuals with prediabetes and early-stage T2D, elevated C-peptide levels generally indicate preserved β-cell function, which is associated with better glycemic control and improved HRQoL [1,11,12,13,14,15]. Conversely, in T1D, where C-peptide is typically absent or minimal, patients often experience increased glycemic variability and dependence on exogenous insulin, which can lower HRQoL [16,17,18,19,20,21]. Notably, studies suggest that even minimal residual C-peptide in T1D may reduce the risk of severe hypoglycemia and improve patient-centered outcomes [22,23]. Despite this evidence, the empirical relationship between C-peptide levels and HRQoL across the continuum of glucose dysregulation remains underexplored.

To address this gap, it is essential to investigate the association between C-peptide levels and HRQoL in individuals across the spectrum of dysglycemia. Understanding this relationship can support a more holistic and individualized approach to diabetes care that considers not only metabolic control but also the broader impacts of the disease on daily life and well-being. Therefore, the primary aim of this study was to assess C-peptide levels and HRQoL in individuals with prediabetes, T1D, and T2D, and to examine their association, with a focus on informing more effective and patient-centered diabetes management strategies.

## 2. Materials and Methods

### 2.1. Participants and Study Design

**Figure A1:**
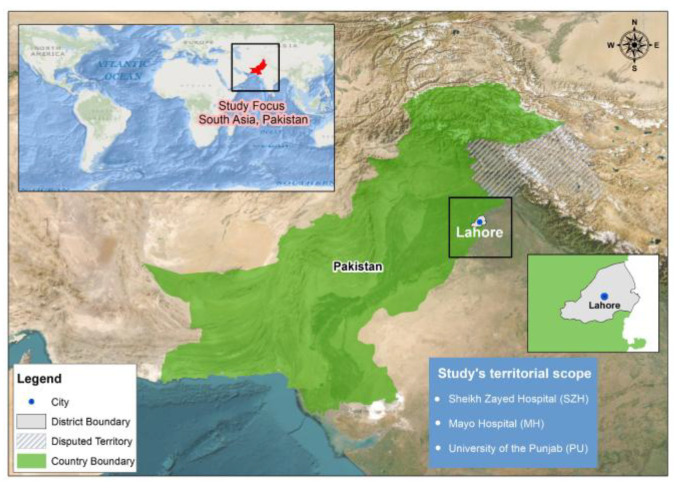
Geospatial representation of the study area.

This study followed an observational cross-sectional design and was conducted at SZH, MH, and University of the Punjab (PU), Lahore, Pakistan. These centers are the most comprehensive private and public tertiary healthcare and education providers operating specialist clinics in diabetology, endocrinology, cardiology, oncology, nephrology, orthopedics, ophthalmology, urology, neurology, internal medicine, family medicine, and general practice. The study’s territorial scope is given in Figure A1. The study participants visiting the outpatient departments (OPDs) were thoroughly informed about the study outcomes, and informed consent was obtained with their signatures or those of their guardians. The study recruitment period was between January 2023 and June 2023. Our study proposal was approved by the Punjab University Institutional Ethics Review Board (PU-IERB) under reference number D/173/FIMS. A prevalence-based sample size formula, that is, n = Z2P(1−P)/d2, was used to calculate the sample size of 301 for the current study, ensuring 80% power at a 95% confidence level (significance level α = 5%), and the diabetes prevalence of 26.7% in the region [24,25]. We recruited 301 individuals who ranged in age from 14 to 65 years and were tested for C-peptide at the hospital following a clinical diagnosis of T1D, T2D, or prediabetes. Prior to testing, all patients were instructed to fast for at least 8 h overnight. Serum C-peptide concentrations were measured using a chemiluminescent immunoassay (ARCHITECT C-Peptide assay, 3L53; Abbott Diagnostics (Chicago, IL, USA)) on the ARCHITECT i2000SR immunoassay analyzer, in accordance with the manufacturer’s instructions. In addition to C-peptide testing, other laboratory evaluations including glycosylated hemoglobin (HbA1c) and glucose were performed at each subsequent visit. Clinical diagnosis of T1D or T2D was made by a clinician based on the following criteria: (a) fasting glucose levels > 7.0 mmol/L, (b) HbA1c > 6.5%, (c) results of autoantibody tests (GAD/anti-islet), and (d) requirement for continuous insulin therapy. Furthermore, trained enumerators conducted face-to-face interviews to collect demographic information, including age, sex, and education level. The interviews were conducted using the EuroQol Group’s Valuation Technology (EQ-VT). HRQoL data were collected using the EQ-5D-5L questionnaire [26], which covers five dimensions: mobility, self-care, usual activities, pain/discomfort, and anxiety/depression. HRQoL scores and the EuroQol Visual Analog Scale (EQ VAS) scores were calculated based on the valuation set provided by Jyani et al. (2022) [27].

**Figure 1:**
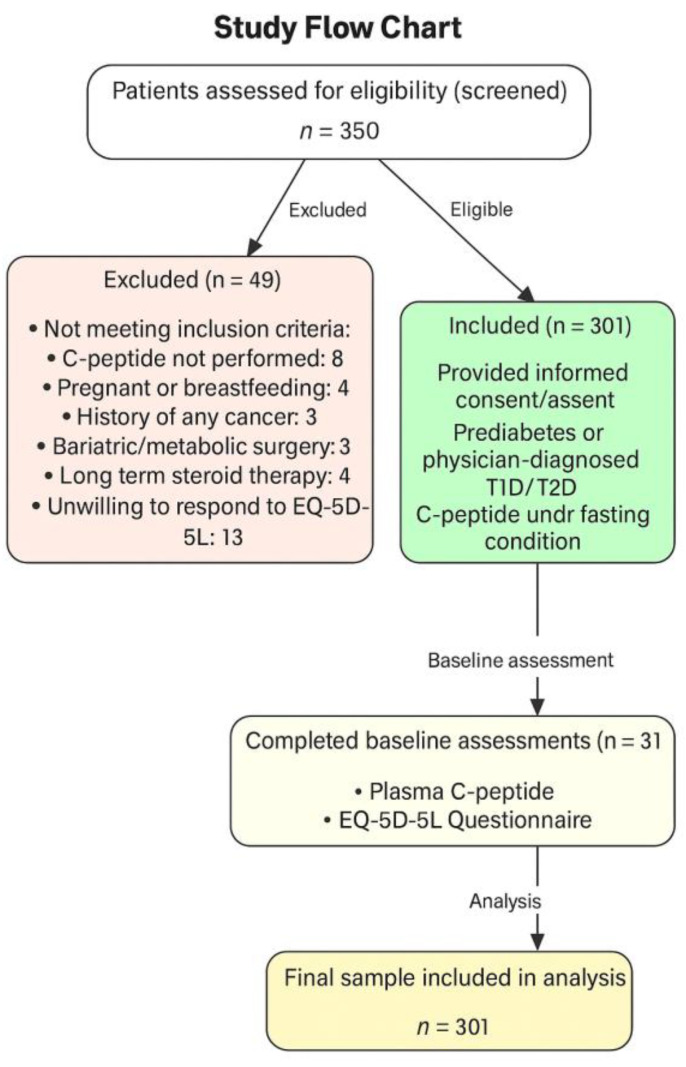
Study flow chart with patients screened, included, and excluded. Values are presented as numbers (n). C-peptide = connecting peptide, T1D = type 1 diabetes, T2D = type 2 diabetes, EQ-5D-5L = EuroQol five dimensions and five levels.

A total of 350 patients were screened during the recruitment period (January to June, 2023). Of these, 49 patients were excluded based on the predefined exclusion criteria (see Section 2.2), including pregnancy or breastfeeding status, history of cancer, recent bariatric or metabolic surgery, long-term steroid use, missing the C-peptide test, or refusal to complete the EQ-5D-5L questionnaire. Finally, 301 patients were included in the analysis. A detailed flow of patient inclusion and exclusion is presented in Figure 1.

Patients were enrolled consecutively as they presented to the outpatient departments and met the eligibility criteria.

### 2.2. Inclusion and Exclusion Criteria

Only patients who were either prediabetic or diagnosed with T1D or T2D by a physician at or before the time of plasma C-peptide examination, and who provided informed consent or had it provided by their guardians, were included in the study. All patients who were pregnant or breastfeeding at the time of the C-peptide test, had a history of cancer, underwent bariatric or metabolic surgery, were on long-term steroid therapy, did not undergo plasma C-peptide testing under fasting or random conditions, or were unwilling to respond to the EQ-5D-5L questionnaire were excluded.

### 2.3. Study Endpoints/Outcome Measures

The primary endpoints of this study were plasma C-peptide levels, HRQoL scores, and EQ VAS scores, as measured using the EQ-5D-5L instrument in individuals diagnosed with prediabetes, T1D, or T2D. A key outcome was to assess the association between plasma C-peptide levels and HRQoL outcomes across these diagnostic categories. Furthermore, this study explored the relationship between C-peptide levels and each of the five specific dimensions of the EQ-5D-5L: mobility, self-care, usual activities, pain/discomfort, and anxiety/depression.

The secondary endpoint was a comparative analysis of overall HRQoL scores between patients with prediabetes, T1D, and T2D.

### 2.4. Statistical Analysis

Data are expressed as mean (SD) where normally distributed and as median (interquartile range [IQR]) where non-normally distributed. Mann–Whitney U and Wilcoxon signed-rank tests were performed to assess the difference in plasma C-peptide and HRQoL scores in prediabetes, T1D, and/or T2D cases. Additionally, the correlation between plasma C-peptide and HRQoL scores was examined using the Spearman rank correlation test. The Kruskal–Wallis test was performed to assess the difference in C-peptide among different levels of problems in mobility, pain/discomfort, usual activities, and self-care, and in anxiety/depression levels. Where the data were missing, mean values were imputed amounting to <2% of the data. Statistical analysis was carried out using R version 4.4.1 (R foundation for Statistical Computing, Vienna, Austria) [28] in RStudio version 2024.04.2+764. The following R packages version 4.4.1 were used: tidyverse, dplyer, and ggplot 2. Significance was assessed at *p* < 0.05 and 95% CI.

## 3. Results

A total of 301 individuals with prediabetes, T1D, or T2D were randomly recruited. Of the total, 42 (14%) had prediabetes (HbA1c between 5.7% and 6.4%), 70 (23.2%) T1D, and 189 (62.8%) T2D.

### 3.1. Patient Characteristics at Baseline

The mean age of the patients was 29.3 + 9.9 years. A total of 178 (59.1%) of the participants were female and 123 (40.9%) were male. The median (IQR) HbA1c and fasting glucose levels were 9.5% (7.3–11.45%) and 10.5 nmol/L (7.1–11.7 nmol/L), respectively. Based on diabetes type, we compared the patient’s demographic, clinical, and laboratory characteristics in Table 1.

### 3.2. Primary Outcome Measures

#### 3.2.1. EQ-5D-5L System

The median (IQR) for the HRQoL score and EQ VAS scores assessed through the EQ-5D-5L instrument were 78.5% (63.2–100%) and 85% (70–90%), respectively. The self-reported HRQoL of the respondents is given in Table 2.

#### 3.2.2. C-Peptide and Its Relation to the HRQoL

**Figure 2:**
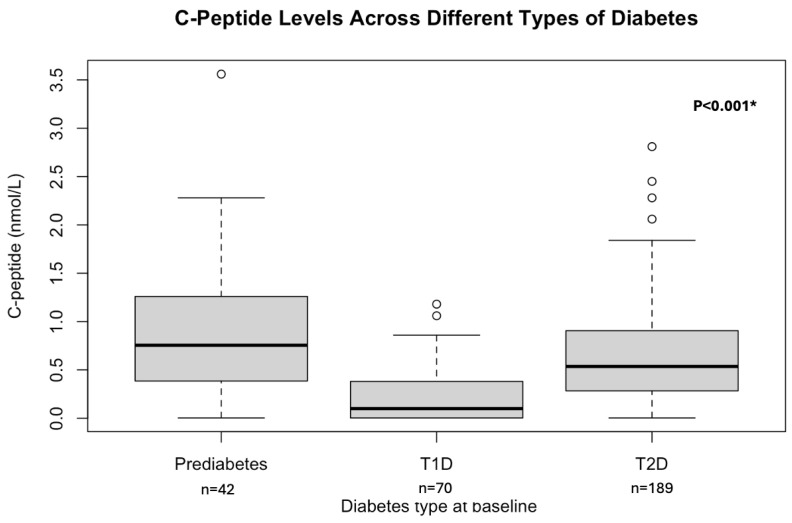
C-peptide levels among different types of diabetes. * Values are presented as numbers (n) and median (IQR). Box plots show median (line), interquartile range (box), whiskers (1.5 × IQR), and outliers (circles) of C-peptide concentrations across participants with prediabetes, T1D, and T2D. Group differences were tested using the Kruskal–Wallis test with Dunn’s post hoc comparisons (Bonferroni correction). C-peptide = connecting peptide; T1D = type 1 diabetes; T2D = type 2 diabetes.

The median (IQR) C-peptide was 0.46 nmol/L (0.13–0.85, nmol/L). We observed significant differences in C-peptide levels among patients with prediabetes, T1D, and T2D; *p*-value < 0.001. Figure 2 will illustrate this difference.

**Figure 3:**
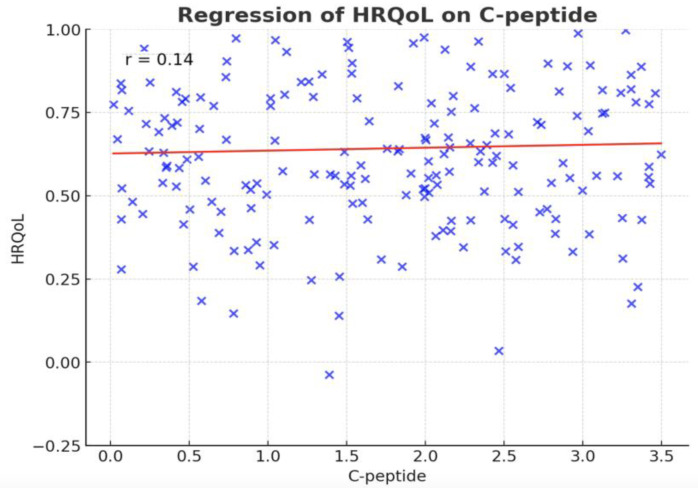
Regression analysis of HRQoL on C-peptide levels. Scatter plot depicting the association between C-peptide levels and HRQoL. A modest but significant positive correlation is observed (r = 0.14, *p* < 0.02). The red line represents the fitted regression line. HRQoL = health-related quality of life, C-peptide = connecting peptide.

**Figure 4:**
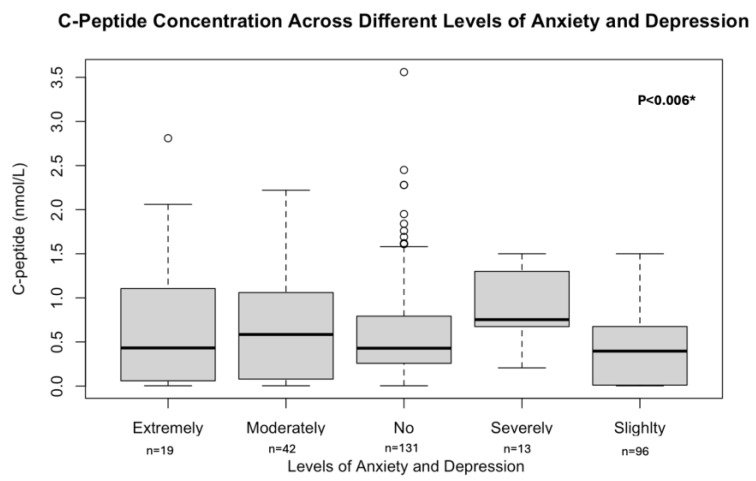
The C-peptide levels among the anxiety/depression levels. * Values are presented as numbers (n) and median (IQR). Box plots show median (line), interquartile range (box), whiskers (1.5 × IQR), and outliers (circles) of C-peptide concentrations across participants with different levels of anxiety and depression (extreme, moderate, none, severe, slight). Group differences were tested using the Kruskal–Wallis test with Dunn’s post hoc comparisons (Bonferroni correction). C-peptide = connecting peptide.

A positive correlation was observed between C-peptide levels and HRQoL in our study (r = 0.14, *p*-value < 0.02). The regression line illustrating this relationship is presented in Figure 3. In addition, C-peptide levels differed significantly in relation to mobility issues, disruptions in usual activities, pain/discomfort levels, and anxiety/depression levels. The Kruskal–Wallis test results showed Chi-square values of 11.75, 13.39, 12.60, and 14.22, with corresponding *p*-values of <0.02, <0.0001, 0.02, and 0.006, respectively. The C-peptide levels can be seen across all the anxiety/depression levels in Figure 4.

### 3.3. Secondary Outcome Measures

#### HRQoL Across Different Types of Diabetes

The HRQoL scores varied significantly among patients with prediabetes, T1D, and T2D, with a Chi-square value of 40.6 and a *p*-value of <0.0001. Additionally, there were notable differences in responses related to mobility issues, self-care difficulties, disruptions in usual activities, pain/discomfort levels, and anxiety/depression levels, with *p-*values of <0.0001, <0.00001, <0.00001, <0.0004, and <0.04, respectively, among the patients with prediabetes, T1D, and T2D. These differences are detailed in Table 3.

## 4. Discussion

Our study offers valuable insights into the HRQoL among individuals with prediabetes, T1D, and T2D using the EQ-5D-5L instrument. The average HRQoL score of 70.4 ± 42.9% suggests a borderline satisfactory level of well-being; however, the substantial variability reflects persistent and multifactorial challenges within this population, highlighting the complex interplay between diabetes type, individual health status, and daily functioning. EQ-5D-5L has been validated in diabetic populations globally—such as in Spain and Singapore—confirming its reliability and sensitivity across dimensions like mobility, self-care, pain/discomfort, and anxiety/depression [29,30,31].

Our results revealed significant differences across specific EQ-5D-5L dimensions, particularly in mobility, self-care, and usual activities, with individuals diagnosed with T1D reporting greater impairments. This aligns with prior work showing that the intensive management regime and risk of complications in T1D can negatively affect both physical and psychological well-being [32,33]. It likely reflects the early onset and treatment demands of T1D, including frequent glucose monitoring and insulin injections.

A key finding from our study is the positive correlation between C-peptide levels and HRQoL. Although this association is statistically significant (r = 0.14, *p* < 0.02), the correlation is weak, suggesting that C-peptide levels alone account for only a small proportion of the variability in patients’ HRQoL and may have limited clinical relevance. Nevertheless, higher C-peptide levels are generally associated with better pancreatic beta-cell function, improved glycemic control, and a lower risk of microvascular complications, all of which can positively impact HRQoL. While direct studies linking C-peptide to HRQoL are limited, the relationship is often inferred through its established connection to diabetes-related complications that adversely affect HRQoL [34,35]. C-peptide, a marker of endogenous insulin production, has been widely studied for its protective effects against microvascular damage and cardiovascular disease in individuals with T2D [36]. In our cohort, individuals with T1D had significantly lower C-peptide levels compared to those with prediabetes or T2D, reflecting reduced beta-cell function. Notably, higher C-peptide levels were associated with better mobility and fewer symptoms of anxiety and depression, suggesting that residual endogenous insulin production may support both physical function and mental well-being [37,38,39,40]. These findings align with experimental evidence showing C-peptide’s protective effects on nerve and kidney function, which could mitigate complications that negatively influence HRQoL [1,15,41]. Therefore, preserving or optimizing residual beta-cell function, particularly in T1D, may not only improve clinical outcomes but also enhance overall patient-reported HRQoL [42,43].

Furthermore, the potential impact of pharmacological interventions on both C-peptide levels and patient HRQoL warrants consideration. Emerging evidence indicates that Sodium–Glucose Co-transporter 2 (SGLT2) inhibitors may enhance pancreatic β-cell function and improve insulin resistance in individuals with type 2 diabetes, suggesting that certain antidiabetic therapies could play a direct role in preserving endogenous insulin production, which has been linked to improved HRQoL [44]. Beyond these physiological benefits, SGLT2 inhibitors have also been associated with greater patient satisfaction, particularly in relation to glycemic control and medication tolerability, as demonstrated in questionnaire-based studies [45]. Integrating these findings into the discussion underscores the importance of considering both clinical efficacy and patient-centered outcomes, thereby offering a more comprehensive perspective on diabetes management.

The use of the EQ-5D-5L tool in our study represents an important methodological contribution. Although widely validated in several countries, including India, Malaysia, and Canada, its application in diabetic populations within Pakistan has been limited [27,46,47]. Our results are consistent with studies such as those by Pongsachareonnont et al. (2024) [48] and Smith et al. (2024) [49], supporting the tool’s relevance across diverse sociocultural settings. Beyond clinical factors, social determinants of health significantly influence HRQoL outcomes. As highlighted in the World Health Organization’s (WHO’s) European Health Equity Status Report, five essential conditions are necessary for sustaining a healthy life: access to quality healthcare, income security and social protection, decent living conditions, social and human capital, and decent work and employment [10]. While access to healthcare services in Pakistan is gradually improving, persistent deficiencies in income security, housing conditions, and employment opportunities likely contribute to the lower HRQoL scores observed in our study. These findings underscore the need for a broader, multisectoral approach to improving well-being among individuals living with diabetes in Pakistan.

Mechanistically, higher C-peptide levels may contribute to improved glycemic stability by reducing the frequency of hypoglycemic and hyperglycemic episodes, both of which are known to exacerbate fatigue, mood fluctuations, disrupted sleep, and diabetes-related emotional distress [1,38]. These effects can significantly undermine HRQoL, especially among individuals with T1D, who typically exhibit lower C-peptide levels. From a clinical perspective, regular monitoring of C-peptide could be a valuable addition to routine diabetes care, helping to identify patients at higher risk of HRQoL deterioration [11,12]. Such assessments would allow for early intervention, not only targeting metabolic control but also addressing physical and psychological aspects of well-being.

To improve outcomes, interdisciplinary strategies that integrate physical rehabilitation, structured self-care education, and mental health support should be prioritized. These approaches can effectively mitigate HRQoL deficits related to mobility, anxiety, depression, and functional limitations. In resource-constrained settings, where healthcare systems face significant limitations, incorporating C-peptide monitoring could also aid in optimizing resource allocation by identifying patients with higher clinical needs [50]. At the same time, broader public health initiatives must address underlying socioeconomic barriers such as unstable employment, poor housing, and limited access to social protection that contribute to poorer diabetes outcomes. Ultimately, tailoring care plans based on C-peptide status and addressing both medical and social determinants of health offers a comprehensive and equitable strategy for enhancing the HRQoL in people living with diabetes [51]. Despite its strengths, this study has some limitations. The cross-sectional design, which captures data at a single time point, limits the ability to establish causal relationships; thus, it remains unclear whether low C-peptide levels lead to poor HRQoL, whether poor HRQoL affects C-peptide levels, or if a third factor underlies the observed association. Additionally, consecutive sampling from tertiary care hospitals may have introduced selection bias by over-representing patients with more severe or complex disease, thereby limiting the generalizability of the findings to broader, community-based populations. Important confounding variables such as socioeconomic status (SES), diabetes duration, comorbidities, and complications were not fully accounted for, all of which are known to independently impact HRQoL. For instance, lower SES is linked to a higher burden of comorbidities and poorer access to healthcare, lifestyle challenges, and psychosocial stressors, which collectively contribute to reduced physical, emotional, and overall HRQoL [52,53]. Diabetes duration, especially in T1D, can also significantly influence both C-peptide levels and long-term HRQoL outcomes. The omission of these confounders may have affected the observed associations. Furthermore, although the EQ-5D-5L is a globally validated tool, its psychometric performance in the Pakistani diabetic population is not yet fully established; hence, cultural variations in HRQoL perceptions should be considered when interpreting the results. Future research employing longitudinal designs with rigorous adjustment for potential confounders is warranted to better elucidate the relationship between C-peptide levels and HRQoL. Such investigations should assess temporal changes in C-peptide concentrations and their associations with insulin resistance, diabetes progression, microvascular complications, and declines in HRQoL. Moreover, the potential role of optimized glucose management strategies in preserving residual β-cell function and consequently enhancing HRQoL merits further evaluation. Addressing these research gaps will provide a more comprehensive understanding of diabetes management, encompassing both clinical outcomes and patient-reported measures.

## 5. Conclusions

In conclusion, both C-peptide levels and HRQoL differ significantly across diabetes types, with lower C-peptide levels associated with reduced mobility, increased pain, and mental health issues. These findings underscore the importance of targeting C-peptide regulation to enhance HRQOL in diabetic populations.

## Figures and Tables

**Table 1:** The demographic, clinical, and laboratory characteristics of the patients.

Characteristics	Prediabetes, n = 42	T1D, n = 70	T2D, n = 189
Age at visit (years) *	36.55 ± 13.4	22.9 ± 7.0	30.3 ± 8.5
Sex male/female *	21 (50%)/21 (50%)	33 (47.2%)/37 (52.8%)	124 (65.6%)/65 (34.4%)
BMI, n = 281 (prediabetes = 42, T1D = 67, T2D = 172) **			
<18.5 kg/m^2^	2 (4.8%)	6 (8.9%)	12 (7%)
BMI 18.5 to <25 kg/m^2^ *	6 (14.3%)	33 (49.2%)	48 (27.9%)
BMI 25 to <30 kg/m^2^	15 (35.7%)	16 (23.8%)	50 (29.1%)
BMI ≥30 kg/m^2^ *	14 (33.3%)	12 (17.9%)	62 (36.0%)
Ever smoked *	8 (19.0%)	8 (11.4%)	29 (16.9%)
SBP (mmHg)	116.5 (110.8–121.8)	114 (107.5–122.5)	120 (111–128)
DBP (mmHg) *	68.5 (63.8–77.5)	67 (61.5–74)	71 (64–80)
C-peptide (fasting), nmol/L *	0.76 (0.39–1.2)	0.09 (0.003–0.38)	0.54 (0.3–0.9)
Hba1C (%) *	5.9 (5.7–6.2)	10.2 (8.3–12.1)	9.9 (8.3–11.9)
Plasma glucose (fasting), nmol/L *	6.5 (5.5–7.6)	12.8 (8.3–16.6)	11.3 (7.8–16.1)

Values are presented as numbers (%), mean ± SD or median (IQR). BMI = body mass index, SBP = systolic blood pressure, DBP = diastolic blood pressure, HbA1C = hemoglobin A1C, C-peptide = connecting peptide, T1D = type 1 diabetes, T2D = type 2 diabetes. * The difference is statistically significant, *p* < 0.05.

**Table 2:** EQ-5D-5L descriptive system of all the participants.

EQ-5D-5L	Mobility (%)	Self-Care (%)	Usual Activity (%)	Pain/Discomfort (%)	Anxiety/Depression (%)
No problem	199 (66.1%)	260 (86.4%)	235 (78.1%)	156 (51.5)	151 (50.2%)
Slight problem	79 (26.2%)	33 (11%)	41 (13.6%)	98 (32.6%)	99 (32.9%)
Moderate problem	13 (4.3%)	5 (1.7%)	22 (7.3)	37 (12.3%)	42 (14%)
Severe problem	6 (2%)	2 (0.7%)	2 (0.7%)	8 (2.7%)	7 (2.3)
Extreme problem	4 (1.3%)	1 (0.3%)	1 (0.3%)	2 (0.7%)	2 (0.7%)
**EQ VAS Score**					
	Mean	SD	Median	25th percentile	75th percentile
EQ VAS Score	0.801	0.128	0.85	0.70	0.90

Values are presented in n (%), SD = standard deviation, EQ-5D-5L = EuroQol five dimensions and five levels.

**Table 3:** Distribution of EQ-5D-5L dimension responses among patients with prediabetes, T1D, and T2D.

HRQoL	Prediabetes (n = 42)	T1D (n = 70)	T2D (n = 189)	All (n = 301)	*p*-Value
Mobility issues	4 (9.5%)	52 (74.3%)	110 (58.2%)	166 (55.1%)	<0.0001 **
Self-care difficulties	4 (9.5%)	45 (64.3%)	106 (56.1%)	155 (51.5%)	<0.00001 **
Disruptions in usual activities	4 (9.5%)	48 (68.6%)	112 (59.3%)	164 (54.5%)	<0.00001 **
Pain/discomfort	4 (9.5%)	53 (75.7%)	113 (59.8%)	170 (56.5%)	<0.0004 **
Anxiety/depression	4 (9.5%)	51 (72.9%)	115 (60.6%)	170 (56.5%)	<0.04 *

Values are presented in n (%), where percentages are based on the total number of participants in each diagnostic group. EQ-5D-5L = EuroQol five dimensions and five levels, HRQoL = health-related quality of life, T1D = type 1 diabetes, T2D = type 2 diabetes. * Significant, ** Highly significant.

## Data Availability

The data presented in this study are not publicly available due to privacy and ethical restrictions involving patient confidentiality. Access to the data may be considered by the corresponding author upon reasonable request and subject to approval by the relevant institutional ethics committee.

## Abbreviations

The following abbreviations are used in this manuscript:

C-peptide	Connecting peptide
T1D	Type 1 diabetes
T2D	Type 2 diabetes
HRQoL	Health-related Quality of Life
WHO	World Health Organization
EQ-5D-5L	EuroQoL Five Dimensions Five Levels
SF-36	Short Form 36 Health Survey
WHOQOL-BREF	World Health Organization Quality of Life-BREF
SZH	Sheikh Zayed hospital
MH	Mayo hospital
PU	University of the Punjab
HbA1c	Hemoglobin A1C
GADA	GAD/anti-islet
EQ VAS	EuroQol Visual Analog
